# Supplementary material for: End Sequence Analysis Toolkit (ESAT) expands the extractable information from single-cell RNA-seq data
Source: Genome Res. 2016 Oct;26(10):1397–410. doi: 10.1101/gr.207902.116 (PMC5052061; doi:10.1101/gr.207902.116)
Supplement: Supplemental Material [file supp_gr.207902.116_Supp_Materials.tar.gz › ESAT-esat_v0.1/doc/html/classbroad_1_1pda_1_1datastructures_1_1_location_aware_matrix.html]

ESAT: broad.pda.datastructures.LocationAwareMatrix Class Reference


ESAT


- Main Page
- Related Pages
- Packages
- Classes
- Files

- Class List
- Class Index
- Class Hierarchy
- Class Members

All Classes Namespaces Files Functions Variables Enumerator Pages

- broad
- pda
- datastructures
- LocationAwareMatrix

Public Member Functions |
Protected Member Functions |
List of all members

broad.pda.datastructures.LocationAwareMatrix Class Reference

Inheritance diagram for broad.pda.datastructures.LocationAwareMatrix:

[legend]

Collaboration diagram for broad.pda.datastructures.LocationAwareMatrix:

[legend]

|  |  |
| --- | --- |
| Public Member Functions | |
|  | LocationAwareMatrix () |
|  | |
|  | LocationAwareMatrix (List< String > rows, List< String > columns, Map< String, LightweightGenomicAnnotation > rowLocations) |
|  | |
|  | LocationAwareMatrix (BufferedReader br, String chr) throws IOException, ParseException |
|  | |
|  | LocationAwareMatrix (List<?extends LightweightGenomicAnnotation > probeData, List< String > samples) |
|  | |
|  | LocationAwareMatrix (MatrixWithHeaders mwh) |
|  | |
| Set< String > | getChromosomes () |
|  | |
| LightweightGenomicAnnotation | getRowPosition (String row) |
|  | |
| void | setColumns (List< String > columns) |
|  | |
| void | setDataDimensions (int rowDim, int columnDim) |
|  | |
| Collection  < LightweightGenomicAnnotation > | getRowAnnotations () |
|  | |
| void | write (BufferedWriter bw) throws IOException |
|  | |
| void | annotation (GenomicAnnotation annotation) |
|  | |
| void | eof () |
|  | |
| void | begin () |
|  | |
| IntervalTree< String > | getRowTree (String chromosome) |
|  | |
| LocationAwareMatrix | submatrixByColumns (Collection< String > columnNames) |
|  | |
| LocationAwareMatrix | submatrixByColumns (String[] columnNames) |
|  | |
| List  < LightweightGenomicAnnotation > | getOverlappers (LightweightGenomicAnnotation annotation) |
|  | |
| void | browserLine (String line) |
|  | |
| void | track (String line) |
|  | |
| LocationAwareMatrix | removeOverlappingRows (LocationAwareMatrix other) |
|  | |
| void | appendRows (LocationAwareMatrix other) |
|  | |
| Map< String,   LightweightGenomicAnnotation > | getRowLocationMap () |
|  | |
| LightweightGenomicAnnotation | getClosest (LightweightGenomicAnnotation annotation) |
|  | |
| LightweightGenomicAnnotation | getPriorClosest (LightweightGenomicAnnotation annotation) |
|  | |
| LightweightGenomicAnnotation | getNextClosest (LightweightGenomicAnnotation annotation) |
|  | |
| Public Member Functions inherited from broad.core.datastructures.MatrixWithHeaders | |
|  | MatrixWithHeaders (String inputFile) throws IOException, ParseException |
|  | |
|  | MatrixWithHeaders (BufferedReader br) throws IOException, ParseException |
|  | |
|  | MatrixWithHeaders (Matrix data, List< String > rowNames, List< String > colNames) |
|  | |
|  | MatrixWithHeaders (Matrix data, List< String > rowNames, List< String > colNames, List< String > rowDescriptions) |
|  | |
|  | MatrixWithHeaders (List< String > rows, List< String > columns, Map< String, String > pidToName) |
|  | |
|  | MatrixWithHeaders (MatrixWithHeaders matrix, List< String > rows, List< String > columns) |
|  | |
|  | MatrixWithHeaders (List< String > rows, List< String > columns) |
|  | |
|  | MatrixWithHeaders (List< String > rows, List< String > columns, List< String > rowDescriptions) |
|  | |
|  | MatrixWithHeaders (String gctFile, String chipFile) throws IOException, ParseException |
|  | |
|  | MatrixWithHeaders (List< List< Double >> rawData, ArrayList< String > rowNameList, List< String > columnNameList) |
|  | |
|  | MatrixWithHeaders (ArrayList< String > rows, ArrayList< String > columns, int DefMatVal) |
|  | |
| Map< String, String > | getPIDToName () |
|  | |
| void | write (BufferedWriter bw) throws IOException |
|  | |
| void | write (String fileName) throws IOException |
|  | |
| void | writeGCT (BufferedWriter bw) throws IOException |
|  | |
| void | writeGCT (String save) throws IOException |
|  | |
| void | writeGCT (String save, Collection< String > columns) throws IOException |
|  | |
| void | writeGCT (String save, Map< String, Collection< String >> experimentInfo, boolean ordered) throws IOException |
|  | |
| void | writeGCT (String save, Map< String, Collection< String >> experimentInfo, Map< String, ExpressionExperimentInfo > fullInfo, boolean info) throws IOException |
|  | |
| void | writeGCT (String save, Collection< String > columns, Collection< String > rows) throws IOException |
|  | |
| void | writeGCTWithHeaders (String save, Map< String, String > columnMapping) throws IOException |
|  | |
| void | writeGCT (String save, Collection< String > columns, Collection< String > rows, int precision) throws IOException |
|  | |
| void | writeGCT (String save, Map< String, String > columns, Map< String, String > rows) throws IOException |
|  | |
| void | writeGCT (String save, Map< String, String > columns, Collection< String > rows) throws IOException |
|  | |
| void | writeGCT (String save, Map< String, String > columns) throws IOException |
|  | |
| void | writeGCT (String save, File chipFile) throws IOException |
|  | |
| Matrix | dataInverse () |
|  | |
| int | columnDimension () |
|  | |
| int | rowDimension () |
|  | |
| double | get (int i, int j) |
|  | |
| double[] | getColumn (int j) |
|  | |
| double[] | getColumn (String columnName) |
|  | |
| double[] | getRow (String rowName) |
|  | |
| void | set (int i, int j, double val) |
|  | |
| void | set (String row, String column, double value) |
|  | |
| void | setRow (String row, double[] vals) |
|  | |
| void | setColumn (double[] vals, String column) |
|  | |
| void | setColumn (double[] vals, int column) |
|  | |
| void | set (String row, int colIdx, double value) |
|  | |
| void | set (int rowIdx, String column, double value) |
|  | |
| double | get (String row, String column) |
|  | |
| boolean | containsColumn (String colName) |
|  | |
| boolean | containsRow (String rowName) |
|  | |
| double | get (String rowName, int colIdx) |
|  | |
| double | get (int rowIdx, String colName) |
|  | |
| List< String > | getColumnNames () |
|  | |
| List< String > | getRowNames () |
|  | |
| String | getRowName (int i) |
|  | |
| String | getColoumnName (int i) |
|  | |
| List< String > | getRowDescriptions () |
|  | |
| boolean | hasColumn (String column) |
|  | |
| boolean | hasRow (String row) |
|  | |
| List< Integer > | getIndecesForRowDescription (String rowDescription) |
|  | |
| void | setRowDescription (int rowIdx, String rowDescription) |
|  | |
| void | setRowDescription (String row, String rowDescription) |
|  | |
| MatrixWithHeaders | filterValuesLargerThanUsingColumn (int colIdx, double maxValue) |
|  | |
| MatrixWithHeaders | filterValuesSmallerThanUsingColumn (int colIdx, double minValue) |
|  | |
| MatrixWithHeaders | filterValuesRangeUsingColumn (int colIdx, double minVal, double maxVal) |
|  | |
| boolean | isColumnNonTrivial (String colName) |
|  | |
| void | compressMatrixByRowDescriptions () |
|  | |
| void | normalizeByMean () |
|  | |
| void | zColumnNormalize () |
|  | |
| void | zRowNormalize () |
|  | |
| void | normalizeBy (double constant) |
|  | |
| void | normalizeColumnsByMean () |
|  | |
| void | normalizeColumnsByMedian () |
|  | |
| MatrixWithHeaders | submatrixByRowDescriptions (Collection< String > rowDescriptions, List< String > newRowNames) |
|  | |
| MatrixWithHeaders | submatrixByRowNames (Collection< String > rowNames) |
|  | |
| MatrixWithHeaders | submatrixByRowNames (String rowName) |
|  | |
| MatrixWithHeaders | submatrixByRowNames (String[] rowNames) |
|  | |
| MatrixWithHeaders | submatrixByColumnNames (Collection< String > columnNames) |
|  | |
| MatrixWithHeaders | submatrixByColumnIndex (Collection< Integer > columnIndex) |
|  | |
| MatrixWithHeaders | submatrixByColumnNames (String[] columnNames) |
|  | |
| MatrixWithHeaders | zScoresByColumn (List< String > controlRows) |
|  | |
| MatrixWithHeaders | tScoresByRow (List< Integer > controlRows, List< Integer > sampleRows, String comparisonName, double fudgeFactor) |
|  | |
| MatrixWithHeaders | rank () |
|  | |
| void | quantileNormalizeColumns () |
|  | |
| MatrixWithHeaders | confidenceByColumn (List< String > controlRows) |
|  | |
| MatrixWithHeaders | sortList (int columnIndex) |
|  | |
| void | append (MatrixWithHeaders other) |
|  | |
| void | appendColumns (MatrixWithHeaders other) |
|  | |
| void | appendRows (MatrixWithHeaders other) |
|  | |
| void | add (MatrixWithHeaders m2) |
|  | |
| void | log2 () |
|  | |
| void | log2 (double fudge) |
|  | |
| void | log10 () |
|  | |
| void | log10 (double fudge) |
|  | |
| void | pow () |
|  | |
| void | round () |
|  | |
| MatrixWithHeaders | times (MatrixWithHeaders other) |
|  | |
| void | minus (MatrixWithHeaders other) |
|  | |
| Matrix | getData () |
|  | |
| Map< String, Collection  < Integer > > | getNanostringReplicateMap () |
|  | |
| Map< String, String > | getNanostringProbeClasses () |
|  | |
| int | getNumberColumns () |
|  | |
| int | getNumberRows () |
|  | |
| Map< String, double[]> | toMap () |
|  | |
| void | writeGMT (String save, double fold) throws IOException |
|  | |
| MatrixWithHeaders | quantileNormalize () |
|  | |
| MatrixWithHeaders | copy () |
|  | |
| MatrixWithHeaders | medianCenterColumns () |
|  | |
| MatrixWithHeaders | medianCenterRows () |
|  | |
| void | medianCenterColumnsThis () |
|  | |
| void | medianCenterRowsThis () |
|  | |
| void | meanNormalizeColumns () throws IllegalStateException |
|  | |
| void | medianNormalizeColumns () throws IllegalStateException |
|  | |
| void | meanNormalizeRows () throws IllegalStateException |
|  | |
| void | medianNormalizeRows () throws IllegalStateException |
|  | |
| MatrixWithHeaders | scaleNorm () |
|  | |
| void | columnDensityNormalization () throws IllegalStateException |
|  | |
| void | rowDensityNormalization () throws IllegalStateException |
|  | |
| void | setPIDToName (Map< String, String > rowDescriptions) |
|  | |
| void | writeCLS (String string, Collection< String > subset, Collection< String > negatives) throws IOException |
|  | |
| void | writeGCTAndCLS (String save, Map< String, ExpressionExperimentInfo > experimentInfo) throws IOException |
|  | |
| void | randomPermuteColumns () |
|  | |
| void | addRow (String row, String description, double[] vals) |
|  | |
| void | addColumn (String column) |
|  | |
| MatrixWithHeaders | filterInvariantGenes (MatrixWithHeaders data, double fold) |
|  | |
| void | writeBox (String save) throws IOException |
|  | |
| MatrixWithHeaders | excludeByRowNames (Collection< String > flaggedGenes) |
|  | |
| double[] | getRow (int i) |
|  | |
| void | floor (double floor) |
|  | |
| void | writeRowNamesToFile (String outFile) throws IOException |
|  | |
| void | writeColoumnNamesToFile (String outFile) throws IOException |
|  | |
| double[] | getMedianOverAllRows () |
|  | |
| double[] | getMeanOverAllRows () |
|  | |
| double[] | getRow (String gene, Collection< String > group1) |
|  | |
| double[] | getValues (String gene, Collection< String > controls) |
|  | |
| boolean | hasNanostringProbeClasses () |
|  | |
| MatrixWithHeaders | multiplyColumnsWithConstants (double[] constants) |
|  | |
| MatrixWithHeaders | multiplyColumnsWithConstants (Map< String, Double > constants) |
|  | |

|  |  |
| --- | --- |
| Protected Member Functions | |
| void | initFromIGVMatrix (BufferedReader br, String header, String chromosomeToLoad) throws IOException, ParseException |
|  | |
| Protected Member Functions inherited from broad.core.datastructures.MatrixWithHeaders | |
|  | MatrixWithHeaders () |
|  | |
| void | initFromReader (BufferedReader br) throws IOException, ParseException |
|  | |
| void | initFromRegularMatrix (BufferedReader br, String header) throws IOException, ParseException |
|  | |
| void | initGCTFromReader (BufferedReader br) throws IOException, ParseException |
|  | |
| void | initFromNanostring (BufferedReader br, String header) throws IOException, ParseException |
|  | |
| void | initNameIndexMaps (List< String > rowNameList, List< String > columnNameList) |
|  | |
| void | setData (Matrix data) |
|  | |
| void | initRowIndexMaps (List< String > rowNameList) |
|  | |
| void | initColIndexMaps (List< String > columnNameList) |
|  | |
| void | initRowDescrIndexMaps (List< String > rowDescriptionList) |
|  | |

## Constructor & Destructor Documentation

|  |  |  |  |  |
| --- | --- | --- | --- | --- |
| broad.pda.datastructures.LocationAwareMatrix.LocationAwareMatrix | ( |  | ) |  |

Here is the caller graph for this function:

|  |  |  |  |
| --- | --- | --- | --- |
| broad.pda.datastructures.LocationAwareMatrix.LocationAwareMatrix | ( | List< String > | *rows*, |
|  |  | List< String > | *columns*, |
|  |  | Map< String, LightweightGenomicAnnotation > | *rowLocations* |
|  | ) |  |  |

Here is the call graph for this function:

|  |  |  |  |
| --- | --- | --- | --- |
| broad.pda.datastructures.LocationAwareMatrix.LocationAwareMatrix | ( | BufferedReader | *br*, |
|  |  | String | *chr* |
|  | ) |  | throws IOException, ParseException |

Here is the call graph for this function:

|  |  |  |  |
| --- | --- | --- | --- |
| broad.pda.datastructures.LocationAwareMatrix.LocationAwareMatrix | ( | List<?extends LightweightGenomicAnnotation > | *probeData*, |
|  |  | List< String > | *samples* |
|  | ) |  |  |

Here is the call graph for this function:

|  |  |  |  |  |  |
| --- | --- | --- | --- | --- | --- |
| broad.pda.datastructures.LocationAwareMatrix.LocationAwareMatrix | ( | MatrixWithHeaders | *mwh* | ) |  |

Here is the call graph for this function:

## Member Function Documentation

|  |  |  |  |  |  |
| --- | --- | --- | --- | --- | --- |
| void broad.pda.datastructures.LocationAwareMatrix.annotation | ( | GenomicAnnotation | *annotation* | ) |  |

Implements broad.core.annotation.AnnotationHandler.

Here is the call graph for this function:

|  |  |  |  |  |  |
| --- | --- | --- | --- | --- | --- |
| void broad.pda.datastructures.LocationAwareMatrix.appendRows | ( | LocationAwareMatrix | *other* | ) |  |

|  |  |  |  |  |
| --- | --- | --- | --- | --- |
| void broad.pda.datastructures.LocationAwareMatrix.begin | ( |  | ) |  |

Implements broad.core.annotation.AnnotationHandler.

|  |  |  |  |  |  |
| --- | --- | --- | --- | --- | --- |
| void broad.pda.datastructures.LocationAwareMatrix.browserLine | ( | String | *line* | ) |  |

Implements broad.core.annotation.AnnotationHandler.

|  |  |  |  |  |
| --- | --- | --- | --- | --- |
| void broad.pda.datastructures.LocationAwareMatrix.eof | ( |  | ) |  |

Implements broad.core.annotation.AnnotationHandler.

|  |  |  |  |  |
| --- | --- | --- | --- | --- |
| Set<String> broad.pda.datastructures.LocationAwareMatrix.getChromosomes | ( |  | ) |  |

|  |  |  |  |  |  |
| --- | --- | --- | --- | --- | --- |
| LightweightGenomicAnnotation broad.pda.datastructures.LocationAwareMatrix.getClosest | ( | LightweightGenomicAnnotation | *annotation* | ) |  |

Here is the call graph for this function:

|  |  |  |  |  |  |
| --- | --- | --- | --- | --- | --- |
| LightweightGenomicAnnotation broad.pda.datastructures.LocationAwareMatrix.getNextClosest | ( | LightweightGenomicAnnotation | *annotation* | ) |  |

Return

Parameters
:   |  |  |
    | --- | --- |
    | annotation |  |

Returns
:   the annotation corresponding to the row that is after the given annotation in chromosome order

Here is the call graph for this function:

|  |  |  |  |  |  |
| --- | --- | --- | --- | --- | --- |
| List<LightweightGenomicAnnotation> broad.pda.datastructures.LocationAwareMatrix.getOverlappers | ( | LightweightGenomicAnnotation | *annotation* | ) |  |

Here is the call graph for this function:

Here is the caller graph for this function:

|  |  |  |  |  |  |
| --- | --- | --- | --- | --- | --- |
| LightweightGenomicAnnotation broad.pda.datastructures.LocationAwareMatrix.getPriorClosest | ( | LightweightGenomicAnnotation | *annotation* | ) |  |

Parameters
:   |  |  |
    | --- | --- |
    | annotation |  |

Returns
:   the annotation corresponding to the row that is before in chromosome order

Here is the call graph for this function:

|  |  |  |  |  |
| --- | --- | --- | --- | --- |
| Collection<LightweightGenomicAnnotation> broad.pda.datastructures.LocationAwareMatrix.getRowAnnotations | ( |  | ) |  |

|  |  |  |  |  |
| --- | --- | --- | --- | --- |
| Map<String, LightweightGenomicAnnotation> broad.pda.datastructures.LocationAwareMatrix.getRowLocationMap | ( |  | ) |  |

|  |  |  |  |  |  |
| --- | --- | --- | --- | --- | --- |
| LightweightGenomicAnnotation broad.pda.datastructures.LocationAwareMatrix.getRowPosition | ( | String | *row* | ) |  |

|  |  |  |  |  |  |
| --- | --- | --- | --- | --- | --- |
| IntervalTree<String> broad.pda.datastructures.LocationAwareMatrix.getRowTree | ( | String | *chromosome* | ) |  |

|  |  |  |  |
| --- | --- | --- | --- |
| void broad.pda.datastructures.LocationAwareMatrix.initFromIGVMatrix | ( | BufferedReader | *br*, |
|  |  | String | *header*, |
|  |  | String | *chromosomeToLoad* |
|  | ) |  | throws IOException, ParseException |

protected

Here is the call graph for this function:

Here is the caller graph for this function:

|  |  |  |  |  |  |
| --- | --- | --- | --- | --- | --- |
| LocationAwareMatrix broad.pda.datastructures.LocationAwareMatrix.removeOverlappingRows | ( | LocationAwareMatrix | *other* | ) |  |

Here is the call graph for this function:

|  |  |  |  |  |  |
| --- | --- | --- | --- | --- | --- |
| void broad.pda.datastructures.LocationAwareMatrix.setColumns | ( | List< String > | *columns* | ) |  |

Here is the call graph for this function:

|  |  |  |  |
| --- | --- | --- | --- |
| void broad.pda.datastructures.LocationAwareMatrix.setDataDimensions | ( | int | *rowDim*, |
|  |  | int | *columnDim* |
|  | ) |  |  |

Here is the call graph for this function:

|  |  |  |  |  |  |
| --- | --- | --- | --- | --- | --- |
| LocationAwareMatrix broad.pda.datastructures.LocationAwareMatrix.submatrixByColumns | ( | Collection< String > | *columnNames* | ) |  |

Here is the call graph for this function:

|  |  |  |  |  |  |
| --- | --- | --- | --- | --- | --- |
| LocationAwareMatrix broad.pda.datastructures.LocationAwareMatrix.submatrixByColumns | ( | String[] | *columnNames* | ) |  |

Here is the call graph for this function:

|  |  |  |  |  |  |
| --- | --- | --- | --- | --- | --- |
| void broad.pda.datastructures.LocationAwareMatrix.track | ( | String | *line* | ) |  |

Implements broad.core.annotation.AnnotationHandler.

|  |  |  |  |  |  |
| --- | --- | --- | --- | --- | --- |
| void broad.pda.datastructures.LocationAwareMatrix.write | ( | BufferedWriter | *bw* | ) | throws IOException |

Here is the call graph for this function:

---

The documentation for this class was generated from the following file:

- java/broad/pda/datastructures/LocationAwareMatrix.java


---

Generated on Fri Aug 15 2014 15:10:59 for ESAT by  

 1.8.7
